# Enhancing the accuracy of knowledge discovery: a supervised learning method

**DOI:** 10.1186/1471-2105-15-S12-S9

**Published:** 2014-11-06

**Authors:** Liangxi Cheng, Hongfei Lin, Feng Zhou, Zhihao Yang, Jian Wang

**Affiliations:** 1 Information Retrieval Laboratory, Faculty of Electronic Information and Electrical Engineering, Dalian University of Technology, Dalian, China

## Abstract

**Background:**

The amount of biomedical literature available is growing at an explosive speed, but a large amount of useful information remains undiscovered in it. Researchers can make informed biomedical hypotheses through mining this literature. Unfortunately, popular mining methods based on co-occurrence produce too many target concepts, leading to the declining relevance ranking of the potential target concepts.

**Methods:**

This paper presents a new method for selecting linking concepts which exploits statistical and textual features to represent each linking concept, and then classifies them as relevant or irrelevant to the starting concepts. Relevant linking concepts are then used to discover target concepts.

**Results:**

Through an evaluation it is observed textual features improve the results obtained with only statistical features. We successfully replicate Swanson's two classic discoveries and find the rankings of potentially relevant target concepts are relatively high.

**Conclusions:**

The number of target concepts is greatly reduced and potentially relevant target concepts gain higher ranking by adopting only relevant linking concepts. Thus, the proposed method has the potential to help biomedical experts find the most useful and valuable target concepts effectively.

## Background

The amount of biomedical literature available is growing at an explosive speed. For example, MEDLINE, a bibliographic database in the field of biomedicine, contains over 16 million references to biomedical journal articles from approximately 5200 biomedical journals worldwide, with more than 2000 completed references added to it each day [1]. Given that people's ability to read substantial amounts of literature is limited and field biomedical experts generally concentrate on relatively narrow topics, the sheer amount of literature available may be a severe challenge for extracting complementary knowledge necessary to the practice of the relevant fields. In this regard, development of a computer-assisted, literature-based approach for mining hidden but central knowledge across disciplines to obtain previously undiscovered public knowledge is of utmost importance.

Swanson [2] initiated literature-based discovery (LBD) research in which two complementary samples of literature are considered: C literature (CL), which describes the C concepts, and A literature (AL), which describes the A concepts (the C and A concepts do not co-occur in any of these two paired literature samples). Meanwhile, B concepts, through which we form the hypothesis that the C and A concepts may have a hidden relation, are described in both CL and AL. Here, we treat CL and AL as non-interactive complementary literature samples and B concepts as linking concepts. Swanson found three linking concepts that led to the discovery of a hidden relation between *Raynaud Syndrome *and *Fish Oil*. Swanson's hypothesis was verified by DiGiacomo et al. [3] in 1989.

One problem in Swanson's method is that it requires a large amount of manual intervention and domain knowledge. In 1997, Swanson and Smalheiser [4] developed an interactive system called Arrowsmith that helps users find potentially meaningful linking concepts between a starting concept and a target concept. However, Arrowsmith only performs in a closed discovery process, which means it does not generate new connections for a given starting concept.

Many researchers have successfully replicated Swanson's discoveries using various approaches. Gordon and Lindsay [5,6] applied several methods of information retrieval, such as token counts, document counts, relative frequencies, and TF*IDF, to LBD. However, the authors exerted much of their effort towards discovering linking concepts instead of target concepts. In addition, their method required further biomedical expertise for application.

Weeber et al. [7] took advantage of a natural language processing tool to identify biomedical concepts and pruned redundant linking concepts and target concepts with use of the UMLS ontology. However, while their method was more automatic than previous works, the authors adopted only a limited number of linking concepts to discover target concepts. They also investigated the potential uses of *thalidomide *[8].

Hristovski et al. [9] applied the association rules to find hidden related Medical Subject Heading (MeSH) concepts through an open discovery approach. In this approach, support and confidence metrics were used to select concepts of interest based on the co-occurrence of MeSH terms.

Srinivasan [10] introduced a method where only the most important linking concepts within certain semantic types were retained to avoid obtaining vast numbers of target concepts. In this method, when the top linking concept is selected from each semantic type, target words gain relatively high rankings. However, when the second or third top linking concepts are selected, the rankings of target words drop.

Cameron et al. [11] adopted the semantic predications along with structured background knowledge and graph-based algorithms to semi-automatically capture the informative associations. They tested and verified Swanson's *Raynaud Syndrome-Fish Oil *hypothesis, demonstrating that Swanson's manually intensive techniques can be undertaken semi-automatically.

Cohen et al. [12] presented the Predication-based Semantic Indexing (PSI), a novel distributional model that encodes predications into a vector space, and provides its possible application to literature-based knowledge discovery. Afterward, Cohen et al. [13] extended the PSI approach to search efficiently across triple-predicate pathways, and utilized it to infer double and triple predicate pathways, which were further adopted to guide search for treatments for other diseases.

## Methods

**Figure 1:**
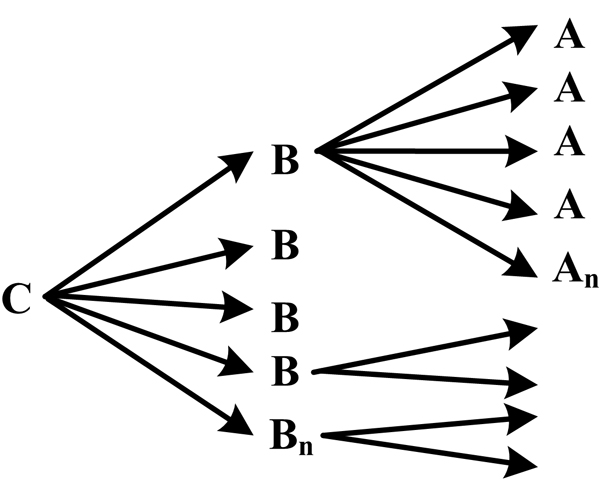
**Open discovery process as defined by Weeber et al**.

Based on Swanson's discovery process, Weeber et al. [7] defined two kinds of knowledge discovery approach, namely, open discovery and closed discovery. An open discovery process is used to generate a hypothesis (Figure 1). For a given starting concept C, concepts that co-occur with C in the literature (called linking concepts B) are found. Concepts that co-occur with linking concepts B (called target concepts A) are then similarly found, bearing in mind that concepts A should not co-occur with the starting concept C. This process can be described as C->B->A.

**Figure 2:**
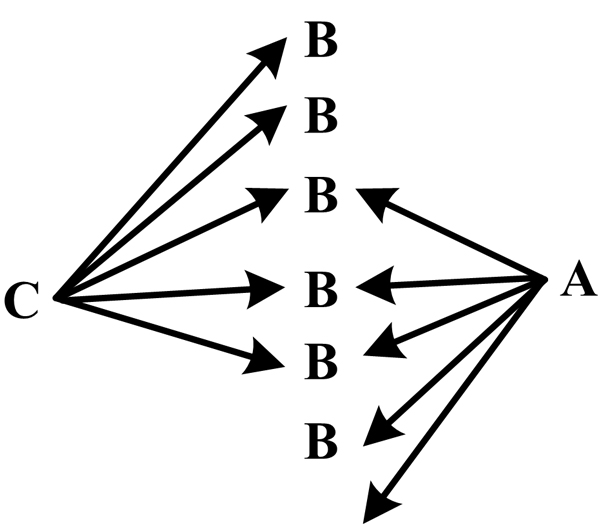
**Closed discovery process as defined by Weeber et al**.

A closed discovery process is used to test a hypothesis (Figure 2). Let's say that, for two given concepts C and A, a researcher would like to find out whether or not hidden links exist between them. The more links found between A and C, the more likely it is that the tested hypothesis is correct. This process can be described as C->B<-A.

**Figure 3:**
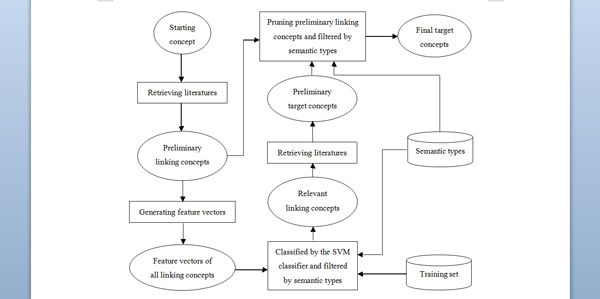
**Framework of our open discovery approach**.

We adopt the open discovery approach to replicate Swanson's discoveries on *Raynaud Syndrome*-*Fish Oil *and *Migraine*-*Magnesium *with starting concepts of *Raynaud Syndrome *and *Migraine*, respectively. The framework of our open discovery approach is illustrated in Figure 3, and 3the detailed processes are described in the following sections.

**Figure 4:**
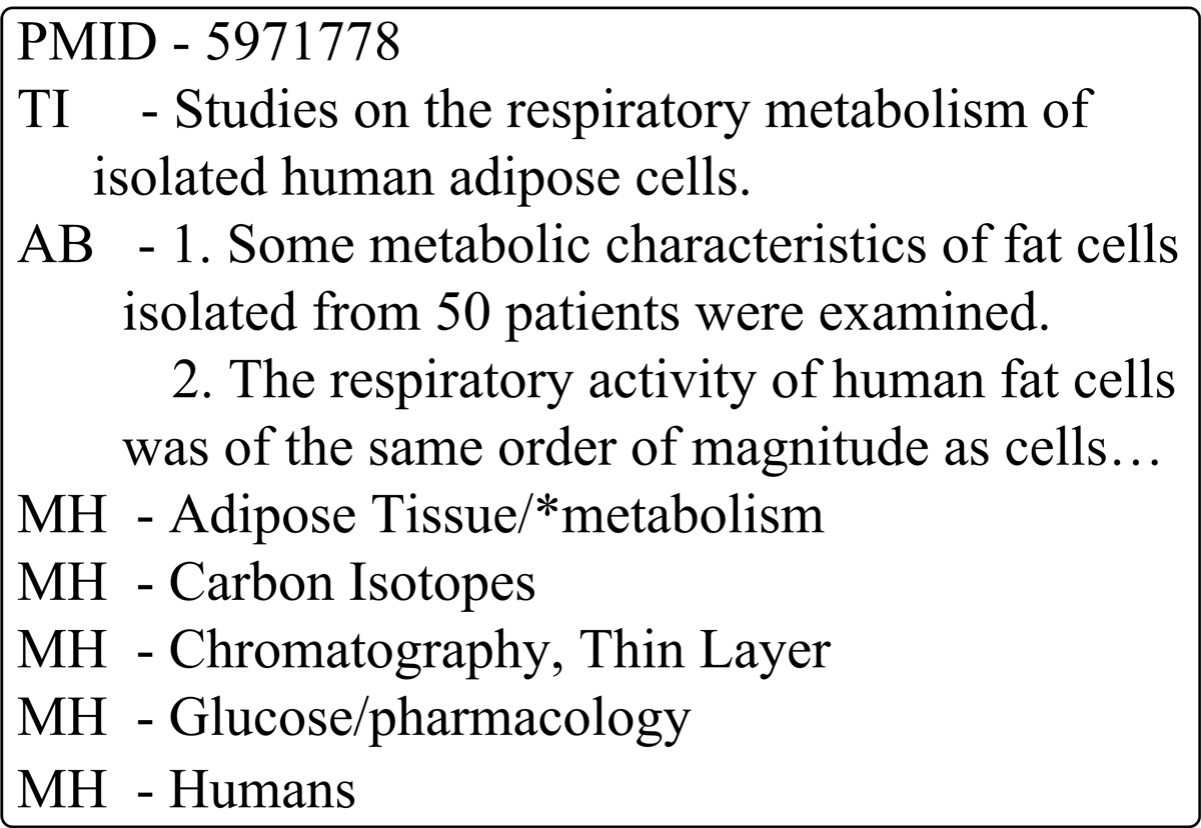
**Brief structure of a MEDLINE record**.

MEDLINE, the primary bibliographic database used in LBD, includes a number of disciplines, such as basic medicine, clinical medicine, experimental medicine, pharmacology, and so on. Figure 4 shows a brief structure of a MEDLINE record, where PMID stands for the unique ID of an article, TI stands for title, AB stands for abstract, and MH stands for MeSH. MeSH, from the National Library of Medicine's controlled vocabulary thesaurus, is selected by experts to index MEDLINE records. Each document is represented by 12 MeSH terms on average; these terms generally consist of terms naming descriptors in a hierarchical structure [14].

### Linking concept selection

For a given starting concept C, all MEDLINE records in the correct time range (discussed in the section of "results and discussion") containing C are retrieved, from which we collect all MeSH terms co-occurring with C as preliminary linking concepts. We find that using only co-occurrence in the MeSH field may be inadequate, since two MeSH terms co-occurring in one MEDLINE record may have no relation. The title and abstract can represent the main idea in biomedical literature. For example, we find over 1800 MeSH terms co-occurring with *Raynaud Syndrome *in the MeSH field but only 700 MeSH terms in the title and abstract fields. If we use all preliminary linking concepts to discover target concepts, the number of target concepts produced will be vast and a large amount of noise will be introduced. Therefore, pruning redundant linking concepts and selecting the most promising ones in open discovery are very important. In this paper, we select linking MeSH terms using both statistical and textual features. Each linking concept is represented by a vector. We use an SVM classifier to classify these linking terms as positive instances (relevant to the starting concept) or negative ones (irrelevant to the starting concept). The SVM classifier is used because results obtained through only co-occurrence are not comprehensive; the SVM classifier can compute a proper weight for each feature and then integrate all of these features.

Here, we use the Semantic Network where all MeSH terms are assigned to at least one of 135 semantic types. For example, the term *Raynaud Syndrome *has semantic type *Disease or Syndrome *and *Fish Oil *has semantic type *Lipid*. We choose the same semantic types used by Weeber et al. [7] and Srinivasan [10]. Thus, the semantic types for linking concepts in the present study are *Biological function, Cell function, Finding, Molecular function, Organism function, Organ or Tissue function, Pathological function, Phenomenon or process*, and *Physiological function*, all of which involve the functions of the disease. The semantic types for target concepts are *Element, Ion, Isotope, Vitamin *and *Lipid*, all of which are dietary factors.

### Statistical features

In the proposed method, we use Mutual Information Measure (MIM) [15], which is widely applied to quantify dependencies between co-occurring concepts, to calculate statistical features. The MIM score is calculated as:

(1) $$MIM\left(A,B\right)={\text{log}}_{2}\left(\frac{P_{AB}}{P_{A}.P_{B}}\right)$$

where *P_AB _*is the joint probability of term A and B co-occurring in the same document, and *P_A_ , P_B _*are the probabilities of observing the term *A, B*, respectively, in any given document. The statistical features consist of two parts: the MIM score calculated for the MeSH field and the MIM score calculated for the title and abstract fields of MEDLINE records.

### Textual features

We select five Boolean features (the value is 1 or 0) to evaluate the degree of connection between two concepts. Taking *Raynaud Syndrome *as an example, its symptoms are *high blood viscosity *and *high platelet aggregation*. Therefore, the interaction words should be "increase" or "aggravate", and so on. Intuitively, two MeSH terms co-occurring in one sentence have a strong connection, so whether or not two MeSH terms co-occur in one sentence of the MEDLINE title and abstract is selected as a Boolean feature. In addition, we establish an interaction word list, which indicates linking concepts may have an influence on the starting concept, to find potentially relevant and promising linking concepts. The interaction word list consists of 115 interaction words and their variants, and the connection is much stronger if two MeSH terms and an interaction word co-occur in one sentence. Therefore, the second Boolean feature we select is whether or not there is an interaction word in the sentence.

In MEDLINE records, some diseases or drug names have abbreviated forms. Usually, at first mention, both the full name and abbreviation appear and only the abbreviation or pronoun is used in subsequent sentences. For example, *AD *is the abbreviation for *Alzheimer's disease*. In the first sentence "*Alzheimer's disease *(*AD*) is the fourth leading cause...," both *Alzheimer's disease *and its abbreviation *AD *appear. In the subsequent sentence "...in the brains of patients with AD are described...," only the abbreviation *AD *appears. Therefore, we select whether or not two MeSH terms co-occur in two neighboring sentences as a Boolean feature and whether or not there is an interaction word as another Boolean feature. Finally, we select whether or not two MeSH terms co-occur in the MEDLINE abstract field as a Boolean feature.

The following are the seven features we select:

I. MIM score calculated for the MeSH field.

II. MIM score calculated for the title and abstract fields.

III. Whether or not two MeSH terms co-occur in one sentence.

IV. Whether or not there is an interaction word in one sentence.

V. Whether or not two MeSH terms co-occur in two neighboring sentences.

VI. Whether or not there is an interaction word in two neighboring sentences.

VII. Whether or not two MeSH terms only co-occur in the MEDLINE abstract field.

In our method, each linking concept is represented by the seven features, and an SVM classifier is employed to classify these linking concepts as positive or negative instances. Positive linking concepts will further be used to discover target concepts.

### Target concept discovery

We search the MeSH field of MEDLINE records for concepts that co-occur with positive linking concepts. These terms are preliminary target concepts. Target concepts that appear in the set of linking concepts are removed and only target concepts belonging to the semantic types *Element, Ion, Isotope, Vitamin *or *Lipid *are retained, namely the final target concepts.

## Results and Discussion

### Datasets

Swanson found that there might be hidden links between *Alzheimer's disease *and *indomethacin *through several linking concepts. In the present paper, we selected linking concepts that had interactions with the starting concept *Alzheimer's disease *as positive instances. Some of these linking concepts were discovered by Swanson and verified to be useful. Other linking concepts were found in the title and abstract fields, which co-occurred in one sentence or two neighboring sentences with the starting concept *Alzheimer's disease *and interaction words. Negative instances included three types: MeSH terms that were too general, MeSH terms that did not co-occur in two neighboring sentences, and MeSH terms that co-occurred in one sentence or two neighboring sentences but contained no interaction word. We then calculated the feature vectors for all positive and negative instances. Since the number of linking concepts that have interactions with the starting concept was very limited, we ultimately obtained 40 positive and 40 negative instances.

For each experiment, we retrieved all linking concepts for the given starting concepts and calculated their feature vectors, which composed the test set. We employed the SVM classifier to tag vectors as positive or negative instances. Next, we made use of the positive instances to discover target concepts.

### Target concept ranking

Pratt and Yetisgen-Yildiz [16] exploited linking term count (LTC) to rank target concepts (i.e., one target concept is more important if there are more linking terms connecting to it). In the proposed method, we adopted a similar idea but did not simply identify concepts co-occurring in the MeSH field as linking terms. Instead, we retrained those "useful" linking terms defined in two ways. First, we identified linking concepts co-occurring with the target concepts in the title and abstract fields as useful linking concepts. Second, we identified linking concept co-occurring with the target concepts and an interaction word in one sentence as useful linking concepts. Using these steps for verification, spurious target concepts can be pruned and the exact text in which linking and target concepts co-occur can be found.

### Performances of features and combinations

The performances of different features and their combinations in *Raynaud Syndrome-Fish Oil *and *Migraine-Magnesium *experiments are shown in Table 1. In this section, selected semantic types were not used for filtering because we intended to examine the effect of our supervised learning method. Given that *Fish Oil *and *Magnesium *are two known effective concepts for *Raynaud Syndrome *and *Migraine*, respectively, linking concepts leading to the target concepts (*Fish Oil *and *Magnesium*) in the MeSH field are considered useful linking terms for ranking target concepts. The percentage of useful LTC is adopted to evaluate the performances of these features and calculated as:

**Table 1:** Performances of different features and their combinations.

Features I	*	*	*	*	*	*	*
Features II		*	*	*	*	*	*
Features VII			*	*	*	*	*
Features V				*	*	*	*
Features VI					*	*	*
Features III						*	*
Features IV							*
percentage of useful LTC (*Raynaud Disease-Fish Oil*)	21.4%	22.9%	22.2%	25.4%	25.5%	27.7%	29.8%
percentage of useful LTC (*Migraine-Magnesium*)	79.7%	82.1%	75.5%	76.6%	77.9%	78.2%	85.6%

Note: "*" stands for including of the corresponding feature.

(2) $$Percentage\text{\_}of\text{\_}Useful\text{\_}LTC=\frac{number\left(Useful\text{\_}Linking\text{\_}Terms\right)}{number\left(All\text{\_}Linking\text{\_}Terms\right)}$$

If spurious linking concepts can be pruned to retain relevant linking concepts leading to the determination of a given target concept, the denominator will decrease and the percentage of useful LTC will increase.

Using only Feature I in the *Raynaud Syndrome-Fish Oil *experiment, the percentage of useful LTC obtained was 21.4%. With the introduction of Feature II, the percentage of useful LTC increased to 22.9%, likely because concepts in the title and abstract fields can reflect the topic more precisely than concepts in the MeSH field. Textual features were added according to the strictness of linking concepts from loose to strong in the order of: VII, V, VI, III, and IV. The percentage of useful LTC decreased slightly when Feature VII was added. However, when the starting and linking concepts were limited to two neighboring sentences, the percentage of useful LTC increased. Interaction words also contributed to the performance of the percentage of useful LTC. After all the textual features were added, the percentage of useful LTC reached a maximum score of 29.8%. As for the ranking of *Fish Oil*, it was not improved as significantly as the percentage of useful LTC. This is probably because the LTC of *Fish Oil *is very limited and there are many general target concepts with a large LTC.

In the experiment of *Migraine-Magnesium*, similar results were obtained, and the percentage of useful LTC increased from 79.7% to 85.6%. *Magnesium *obtained a No. 1 ranking compared with Pratt and Yetisgen-Yildiz's No. 11 [16].

From Table 1 we can draw some conclusions: 1. Feature II (MIM score calculated for the title and abstract fields) can better reflect the dependencies of two concepts than Feature I (MIM score calculated from the MeSH field). 2. Textual features improve the results obtained with only statistical features. 3. Interaction words contribute to the performance of the percentage of useful LTC.

Since incorporating statistical and textual features can increase the percentage of useful linking concepts and retain relevant linking concepts, we will use these seven features to represent linking concepts in the following experiments.

#### Raynaud Syndrome-Fish Oil

In the *Raynaud Syndrome-Fish Oil *experiment, we utilized MEDLINE records obtained prior to 1986 since Swanson [17] made the discovery in this year. The starting concept C *is Raynaud Syndrome*. We retrieved all MeSH terms co-occurring with C and generated their feature vectors. Then, we employed the SVM classifier to classify them as positive or negative instances.

As can be seen from Table 2 rankings of *Fish Oil *and percentages of useful linking concepts obtained using positive concepts are much higher than those using all linking concepts. By using positive concepts, many irrelevant linking concepts were excluded, which would otherwise generate many target concepts irrelevant to the starting concept. The LTC of target concepts discovered by these negative linking concepts decreased after pruning, and therefore the ranking of relevant target concepts such as *Fish Oil *were promoted. Further filtering with the semantic types pushes the ranking of *Fish Oil *even higher.

**Table 2:** Experimental results of *Raynaud Syndrome-Fish Oil *problem.

Ranking rules	Linking concepts	LTC	Useful LTC	Percentage of useful LTC	Ranking of *Fish Oil*
Rule 1	all	1852	148	8.0%	141
	Positive	323	49	15.2%	121
	all + ST	176	15	8.5%	128
	positive + ST	47	7	14.9%	80
Rule 2	All	1852	68	3.7%	128
	Positive	323	28	8.7%	105
	all + ST	176	8	4.5%	96
	positive + ST	47	6	12.8%	45

Note: "useful LTC" stands for the number of concepts leading to the finding of *Fish Oil*; "ST" stands for semantic types filtering.

As aforementioned in the subsection of "target concept ranking", two kinds of "useful" linking terms for ranking target concepts are defined: linking concept co-occurring with the target concepts in the title and abstract fields (Rule 1) and linking concept co-occurring with the target concepts and an interaction word in one sentence as useful linking concepts (Rule 2). The latter is stricter so that more precise but less useful linking concepts are retained and, as experimental results show, the rankings of *Fish Oil *increase and the percentages of useful linking concepts decrease.

*Fish Oil *obtained the best ranking (No. 45) by using positive concepts and semantic type filtering in a sentence-level experiment.

Table 3 shows part of the target concepts discovered at the sentence level. Weeber et al. [7] used three pathways (*Platelet Aggregation, Blood Viscosity*, and *Vascular reactivity*) identified by Swanson to discover target concepts. In their experiments, *Fish Oil *ranked 7 and 20 for the pathways *Platelet Aggregation *and *Blood Viscosity*, respectively. The pathway *Vascular reactivity *could not find *Fish Oil*. In contrast, when all linking concepts were used, as in our experiment, *Fish Oil *ranked 45 in all target semantic types. Srinivasan [10] ranked the target concepts in each target semantic type and *Fish Oil *ranked 19 under the semantic type *Lipid*; in our experiment, it ranked 16.

**Table 3:** Part of target concepts discovered for *Raynaud Syndrome*

Target concept	Ranking in target semantic types	Ranking in semantic type *Lipid *
Carbon	1	
Fatty Acids	2	1
Nitrogen	3	
Oil	7	2
...	...	...
Cadmium	26	...
Platelet Activating Factor	27	7
Lipoproteins, VLDL	28	8
...	...	
Liposomes	32	9
Oleic Acid	33	10
Lipoproteins, LDL	34	11
Lipoproteins, HDL	35	12
...	...	...
Phosphatidylcholines	43	15
Anions	44	
Fish Oil	45	16
...	...	...

Swanson found three linking concepts through which he discovered the hidden relation between *Raynaud Syndrome *and *Fish Oil*. These linking concepts are *Vasoconstriction, Blood Viscosity*, and *Platelet Aggregation*. As shown in Table 4 the proposed method was able to classify these concepts as positive instances and adopted them to discover target concepts further. Meanwhile, the number of linking concepts was reduced from 1852 to 323.

**Table 4:** Comparison of useful linking concepts between Swanson's and our system for the *Raynaud Syndrome-Fish Oil *problem

Three useful linking concepts discovered by Swanson	Discovered by our system
Vasoconstriction	Y
Blood Viscosity	Y
Platelet Aggregation	Y

#### Migraine-Magnesium

In the *Migraine-Magnesium *experiment, we utilized MEDLINE records prior to 1988 since Swanson [18] made the discovery in this year. The starting concept C adopted was *Magnesium*.

Using positive instances to discover target concepts, *Magnesium *obtained a high ranking. Further filtering with the semantic types pushed *Magnesium *to rank No. 1. Meanwhile, the percentage of useful linking concepts improved. Table 5 shows the results obtained by different experimental methods. In these experiments, the ranking of *Magnesium *was relatively high and consistently ranked No. 1.

**Table 5:** Experimental results of *Migraine-Magnesium *problem.

Ranking rules	Linking concepts	LTC	Useful LTC	Percentage of useful LTC	Ranking of *Magnesium*
Rule 1	All	2898	1405	48.5%	2
	positive	529	398	75.8%	1
	all + ST	236	199	84.3%	2
	positive + ST	61	53	86.9%	1
Rule 2	All	2898	827	28.5%	4
	positive	529	290	54.8%	2
	all + ST	236	80	33.9%	5
	positive + ST	61	39	63.9%	1

Note: "useful LTC" stands for the number of words leading to the finding of *Magnesium*; "ST" stands for semantic type filtering.

Table 6 shows part of the target concepts we discovered at the sentence level. In Srinivasan's method [10], the best rankings obtained for *Magnesium *were 5 and 12 when linking terms ranking highest and second highest within each semantic type were adopted. In Pratt and Yetisgen-Yildiz's method [16], the LTC was 29 and the best ranking of *Magnesium *was 11. In our experiment, *Magnesium *obtained a rank of No. 1 with an LTC of 39.

**Table 6:** Part of top ranking target concepts discovered for *Migraine*

Target concept	LTC	Ranking in target semantic types	Ranking in semantic type *Element, Ion*, or *Isotope *
Carbon	39	1	1
Magnesium	39	1	1
Ions	37	2	2
Nitrogen	34	3	3
Oil	32	4	
Hydrogen	31	5	4
Fats	28	6	5
Iodine	24	7	6
...	...	...	...

Table 7 lists eleven complementary arguments that connect *magnesium deficiency *with *migraine*, as discovered by Swanson et al. [19]. The proposed method was able to classify 9 of these arguments as positive instances. Although the proposed method missed two useful linking concepts, it reduced the number of linking concepts from 2898 to 529.

**Table 7:** Comparison of useful linking concepts between Swanson's and our system for the *Migraine-Magnesium *problem

Eleven useful linking concepts discovered by Swanson	Discovered by our system
Stress	Y
Vascular Tone	Y
Calcium Channel Blockers	Y
Spreading Cortical Depression	N
Epilepsy	Y
Platelet Aggregation	Y
Serotonin	Y
Substance P	Y
Prostaglandins	Y
Inflammation	Y
Hypoxia	N

## Conclusions

In the field of literature-based hidden knowledge discovery, popular methods based on co-occurrence produce too many target concepts, leading to the declining ranking of potentially relevant target concepts. In the current paper, we propose a new method for choosing useful and promising linking concepts. This method selects statistical and textual features and employs an SVM model to classify linking concepts whether relevant to the starting concepts. Linking concepts classified as relevant to the starting concept are adopted to further find target concepts.

The current experimental results on two classic experiments, *Raynaud Syndrome*-*Fish Oil *and *Migraine*-*Magnesium*, show that the ranking of potentially relevant target concepts is promoted by adopting only relevant linking concepts. In addition, we employ the percentage of useful LTC to evaluate the performance of the proposed method. The results show that the percentage of useful LTC is significantly improved. The proposed system is more automatic than other methods, with only required manual step of the training set construction. Thus, the proposed method has the potential to help biomedical experts find the most useful and valuable target concepts effectively. In future research, we aim to find more useful linking concept features to gain better results.

## Competing interests

The authors declare that they have no competing interests.

## Authors' contributions

LC participated in the design of the study, helped to carry out the study and with the statistical analysis, and drafted the manuscript. HL designed the study and performed the statistical analysis. FZ carried out the study and participated in its coordination. ZY conceived of the study and helped to draft the manuscript. JW helped to carry out the study and to draft the manuscript. All authors read and approved the final manuscript.

## Declarations

Publication of this article has been funded by the Natural Science Foundation of China (No. 60673039, 60973068, 61277370, 61070098, 61272373), the National High Tech Research and Development Plan of China (No. 2006AA01Z151), Natural Science Foundation of Liaoning Province, China (No. 201202031), State Education Ministry and The Research Fund for the Doctoral Program of Higher Education (No. 20090041110002).

This article has been published as part of *BMC Bioinformatics *Volume 15 Supplement 12, 2014: Selected articles from the IEEE International Conference on Bioinformatics and Biomedicine (BIBM 2013): Bioinformatics. The full contents of the supplement are available online at http://www.biomedcentral.com/bmcbioinformatics/supplements/15/S12.
